# Editorial: New developments in Agrobacterium Mediated Transformation of tree fruit crops, volume II

**DOI:** 10.3389/fpls.2023.1249563

**Published:** 2023-07-12

**Authors:** Vladimir Orbovic, Humberto Prieto

**Affiliations:** ^1^ Citrus Research and Education Center, Institute of Food and Agricultural Sciences (IFAS), University of Florida, Lake Alfred, FL, United States; ^2^ Biotechnology Laboratory, La Platina Station, Instituto de Investigaciones Agropecuarias, Santiago, Chile

**Keywords:** genetic transformation, Agrobacterium tumefaciens, fruit crops, tree, methodology

The Agrobacterium-Mediated Transformation has been used for genetic engineering of Tree Fruit Crops for more than three decades. Much progress has been made in this field (Song et al., 2019) although many obstacles remain for utilization of this technology at levels used for herbaceous and annual plants. In the second volume of this Research Topic, there are papers describing approaches different research groups are taking to facilitate genetic transformation of recalcitrant tree species and on a more basic level understand mechanisms of insertion of T-DNA into the genome of host cells.

In an elegant study Gelvin et al. investigated formation of T-circles as a proxy for understanding T-DNA integration. In this work, regions associated with the LB-RB junction were characterized in detail from T-circles formed in transgenic plants of *Nicotiana benthamiana* or *Arabidopsis thaliana*. It was shown that RB-LB junctions in T-circles were similar to junctions between T-DNA and the plant DNA where integration took place. Similarities included: higher frequency of deletions and more extensive changes in sequence at the LB in comparison to RB; presence of microhomology at junction sites; presence of filler DNA originating from Agrobacterium or plant genome; concatemeric organization of multiple copies of T-DNA with RB-RB and LB-LB junctions being more frequent than RB-LB junctions. Furthermore, the authors have shown that T-circles formation proceeded without Ku80 and ω mutation in VirD2 gene of Agrobacterium had an effect similar to the effect exerted on T-DNA integration. Based on their data the authors suggested that formation of T-circles can be used to study all aspects of T-DNA integration into host’s genome.

Most of the work published on citrus transformation, utilized material from only a few cultivars that were relatively easy to transform (Song et al, 2021). Mandadi’s group at TAMU (Dominguez et al.) developed a method that should facilitate transformation of 14 citrus cultivars. They have achieved this by addition of supplements such as spermidine, and lipoic acid to media used in transformation protocol and by using a helper plasmid pCH32 containing additional copies of VirG and VirE genes. Presumed role of the supplements was to lessen the oxidative stress in explants incubated with Agrobacterium whereas VirG and VirE genes were supposed to enhance integration of T-DNA. Transformation efficiency for some cultivars was increased to 11% (as percentage of GUS-positive shoots produced per number of explants used in the experiment) and the period for production of soil-acclimated transgenic plants was shortened to six months for some cultivars. Based on Southern blot analyses, the number of integrated T-DNA copies ranged from one to four. This method has a potential to help researchers using Agrobacterium-mediated transformation achieve higher productivity.

Researchers working with apples face similar challenges as those working on citrus transformation include low efficiency. In the review paper (Schropfer et al.) summarize the progress in application of transgenic technology to apples and offer possible directions for the future research. The non-browning Arctic^®^ apples remain a single achievement of research in apples where final product found its way to the market. However, in recent years use of various starting materials and media for transformation, refinement of agroinfiltration conditions, as well as pre-transformation with a BABY BOOM transcription factor led to improved transformation efficiency in apple. New strategies for gene silencing have also been used for apples. Those include RNAi-based silencing by stable transformation with hairpin gene constructs, virus-induced gene silencing (VIGS) and artificial micro RNAs (amiRNAs). The targeted genome editing (GE) in apples resulted in production of a homohistont GE line into which a biallelic mutation was specifically inserted in a target gene. Finally, this review discusses methods in which genetically modified plants are used for the rapid crop cycle breeding system.

Anthocyanin biosynthesis in the peal of red pear (*Pyrus pyrifolia* Nakai.) is regulated by complex interaction of multiple transcriptional regulators where BBX (B-box) plays the important role. However, the upstream regulation of BBX genes has not been well characterized. It was known from recent work that PpBBX18 interacts with PpHY5 *via* two B-box domains and then forms a heterodimer. The PpHY5 binds to the G-box motif of PpMYB10 and PpBBX18 provides the trans-acting activity, thus inducing transcription of PpMYB10 as well as anthocyanin biosynthesis (Bai et al., 2019). In the work by Zhang et al. PpZAT5, a cysteine2/histidine2-type transcription factor, was discovered as the upstream negative regulator of PpBBX18. PpZAT5 expression was inhibited by light, which is converse to the expression pattern of anthocyanin-related structural genes. The results of this study showed that PpZAT5 functions as a transcriptional repressor which can directly bind to the CAAT motif of PpBBX18 and inhibit its expression thereby inhibiting anthocyanin biosynthesis.

Genetic transformation of grapevine cultivars can be considered extremely difficult. Capriotti et al. have combined somatic embryogenesis and organogenesis to facilitate transformation of the Vitis vinifera cultivars Ancellotta and Lambrusco Salamino, in comparison with the model cultivar Thompson Seedless. Plantlets obtained through from somatic embryogenesis from flowers were used as starting materials to obtain explants for transformation experiments. Cotyledons and hypocotyls were incubated on media with different combination of growth regulators to estimate their competence for shoot organogenesis. While explants from both Ancellotta and Lambrusco Salamino produced high number of GFP-expressing calli, this approach has produced only one transgenic plant of the Ancelloto cultivar. Definition of novel protocols combining *in vitro* regeneration and genetic transformation of grapevine including the wine grape and table grape varieties will be essential for the application of emerging methods aimed at improvement of agronomic and commercial traits.

## Author contributions

VO wrote the Editorial. HP read and approved the text.

